# Site‐Specific Wetting of Iron Nanocubes by Gold Atoms in Gas‐Phase Synthesis

**DOI:** 10.1002/advs.201900447

**Published:** 2019-05-02

**Authors:** Jerome Vernieres, Stephan Steinhauer, Junlei Zhao, Panagiotis Grammatikopoulos, Riccardo Ferrando, Kai Nordlund, Flyura Djurabekova, Mukhles Sowwan

**Affiliations:** ^1^ Nanoparticles by Design Unit Okinawa Institute of Science and Technology (OIST) Graduate University 1919‐1 Tancha Onna‐son 904‐2151 Okinawa Japan; ^2^ Department of Physics and Helsinki Institute of Physics University of Helsinki P.O. Box 43 FI‐00014 Helsinki Finland; ^3^ Dipartimento di Fisica Universita di Genova Via Dodecaneso 33 I16146 Genova Italy

**Keywords:** Fe–Au nanoparticles, growth kinetics, inert gas condensation, metastability, wetting

## Abstract

A key challenge in nanotechnology is the rational design of multicomponent materials that beat the properties of their elemental counterparts. At the same time, when considering the material composition of such hybrid nanostructures and the fabrication process to obtain them, one should favor the use of nontoxic, abundant elements in view of the limited availability of critical metals and sustainability. Cluster beam deposition offers a solvent‐ and, therefore, effluent‐free physical synthesis method to achieve nanomaterials with tailored characteristics. However, the simultaneous control of size, shape, and elemental distribution within a single nanoparticle in a small‐size regime (sub‐10 nm) is still a major challenge, equally limiting physical and chemical approaches. Here, a single‐step nanoparticle fabrication method based on magnetron‐sputtering inert‐gas condensation is reported, which relies on selective wetting of specific surface sites on precondensed iron nanocubes by gold atoms. Using a newly developed Fe–Au interatomic potential, the growth mechanism is decomposed into a multistage model implemented in a molecular dynamics simulation framework. The importance of growth kinetics is emphasized through differences between structures obtained either experimentally or computationally, and thermodynamically favorable configurations determined via global optimization techniques. These results provide a roadmap for engineering complex nanoalloys toward targeted applications.

## 1 Introduction

Hybrid multicomponent nanoparticles with tailored morphologies and chemical compositions have received significant attention due to their unique properties. In particular, the shape control of phase‐separated bi‐metallic nanoparticles has been widely explored, as it can lead to highly advantageous characteristics in applications such as heterogeneous catalysis,^1^, ^2^, ^3^ plasmonics,^4^, ^5^, ^6^ or biosensing.^7^, ^8^ The rational design and synthesis of optimized nanoparticles requires advanced growth techniques with superior control over thermodynamic as well as kinetic parameters.^9^ Among the variety of methods available to date, magnetron‐sputtering inert gas condensation enables the versatile and flexible synthesis of size‐ and shape‐controlled nanoparticles,^10^, ^11^, ^12^ providing opportunities to achieve metastable configurations due to fast nonequilibrium growth kinetics.^13^, ^14^

In this article, we report the gas‐phase synthesis of phase‐separated Fe–Au nanoparticles with sophisticated cuboid morphologies and explain the counterintuitive growth mechanism with a detailed model at the atomic scale. This material system is particularly promising for the realization of hybrid nanoparticles incorporating both magnetic and plasmonic properties, which is of high relevance for targeted drug delivery,^15^ multimodal bioimaging,^16^, ^17^ and biomarker diagnostics.^18^ Previous reports on Fe–Au nanoparticles realized by physical deposition methods include core–shell morphologies obtained through thermal treatments and segregation^19^, ^20^ as well as alloy nanoparticles realized by magnetron‐sputtering inert gas condensation.^21^, ^22^ Here, we demonstrate that coalescence of preformed Fe and Au monometallic clusters during gas‐phase synthesis triggers distinct surface wetting events that lead to complex morphologies, i.e., from Fe cubes vertex to vertex/edge‐decorated by Au, and occasionally accompanied by multiple embedded frame‐like structures. The growth mechanism is explained by atomistic calculations explicitly designed to reflect the experimental conditions. To this end, a new interatomic potential for Fe and Au interactions was specifically developed; the two elements, a typical body‐centered cubic transition metal and a face‐centered cubic noble metal, respectively, had been successfully modeled in the past, but using force fields of inherently different formalisms which could not be readily combined to define mixed‐element, cross‐potential terms.^23^, ^24^ Only very recently a dedicated semi‐empirical potential was reported which reproduced similar alloying behavior as the potential reported here.^25^ Our results, exhibiting excellent agreement between experiments and molecular dynamics (MD) modeling, provide valuable guidance for controlling nanoparticle shapes with increasing complexities during gas‐phase synthesis, while at the same time advancing the understanding of the underlying growth mechanisms.

## 2 Results and Discussion

### 2.1 Overview

The Fe–Au nanocubes were synthesized using a magnetron‐sputtering inert gas condensation system^10^ relying on co‐deposition^26^ from three independent targets that can be controlled individually (two 1'' Fe targets, one 1'' Au target). Three different samples were investigated: i) a low Au concentration sample, ii) a pure Fe sample, and iii) a high Au concentration sample. Further details on the synthesis conditions are reported in the Section 1.1 of the Supporting Information.

#### 2.1.1 Synthesis and Characterization of Low Au Concentration Sample

**Figure 1:**
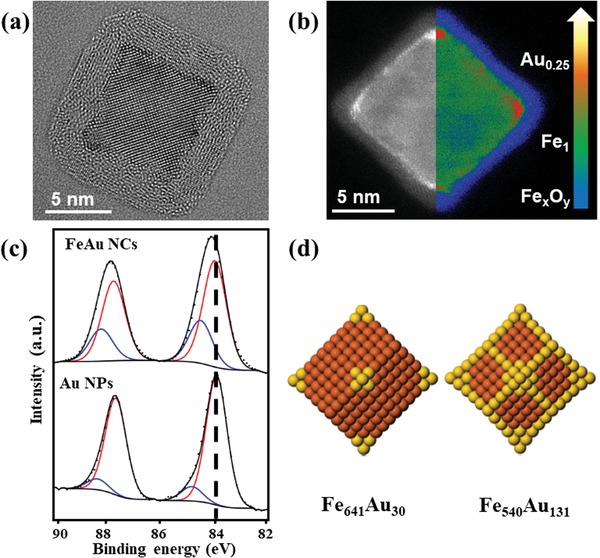
Structure and chemical composition of the low Au concentration sample. a) High‐resolution transmission electron micrograph of Fe–Au nanocube. b) High‐angle annular dark‐field image of a Fe–Au nanocube, showing Au being present at the vertices and edges. Left: STEM image using black/white color coding with bright contrast corresponding to the higher atomic numbers (Au); Right: qualitative false color image showing the Fe oxide (Fe*_x_*O*_y_* in blue) shell, the metallic Fe (Fe_1_ in green) core, and the Au‐decorated vertices (Au_0.25_ in orange, 25 at.% Au obtained by EELS quantification). c) X‐ray photoelectron spectrum of Fe–Au nanocube sample (top) and Au nanoparticle reference sample (bottom). d) Atomistic global optimization calculations for nanoclusters of the Fe–Au system, clearly indicating vertex and edge decoration by Au atoms.

A high‐resolution transmission electron micrograph (HRTEM) of a representative low Au concentration Fe–Au nanocube is presented in **Figure** **1**a. The nanoparticle core exhibits body‐centered cubic structure and, due to ambient air exposure, is covered by an Fe oxide shell (*γ‐*Fe_2_O_3_ and/or Fe_3_O_4_, denoted as Fe*_x_*O*_y_* henceforth) with an epitaxial relationship to the core, similar to pure Fe nanocubes reported before.^27^, ^28^, ^29^ Additional TEM micrographs and corresponding nanoparticle size distributions are shown in Figure S2 in the Supporting Information. To investigate the Au distribution within the nanoparticles, we used high‐angle annular dark‐field scanning transmission electron microscopy (HAADF‐STEM) combined with energy electron loss‐spectroscopy (EELS). The HAADF‐STEM image contrast corresponds to differences in the atomic number Z, and reveals a brighter core compared to the surrounding shell, which confirms the Fe core/Fe oxide shell morphology (see Figure 1b). In addition, the image contrast suggests the presence of Au at the cube vertices and edges of the metallic Fe core. The Au content on the different locations is highlighted by the false color image in Figure 1b (right) and clearly shows the Au enrichment of the vertices (and, to a lesser extent, the edges), while the core consists of metallic Fe and the shell of Fe oxide. Multiple EELS point‐scans on different nanoparticles and various positions (core, vertices, and edges) corroborated this finding for both the low and high Au concentration samples (see Figure S3 in the Supporting Information). The morphology of the presented Fe–Au nanocubes is in clear contrast to previously reported alloyed FeAu nanocubes that were obtained through magnetron‐sputtering of a composite Fe target with inserted Au pellets,^22^ foreshadowing the importance of kinetics effects upon particle formation, due to the different deposition set‐up.

Moreover, using X‐ray photoelectron spectroscopy (XPS) we observed different Au coordination for both deposition setups, compared with an Au reference sample fabricated with our deposition system, as shown in Figure 1c (see also Figure S4 in the Supporting Information). In the present Fe–Au nanocubes, Au atoms are primarily found in Au–Au coordination^30^ (peak at 83.9 eV) similar to the Au reference sample (84 eV), which corroborates phase separation rather than alloying, accompanied by elemental enrichment. The smaller peak located at 84.8 eV can be attributed to the contribution of small Au clusters which have been reported to present a positive shift in binding energy compared with the bulk value.^31^ In contrast, the XPS spectra for the aforementioned composite target case show a negative shift in the binding energy (0.6 eV) which is attributed to Fe–Au alloy formation^32^ (see Figure S4 in the Supporting Information). Additionally, the ratio between the peak areas of metallic Au 4f (83.9 eV) and Fe 2p ((706.3 eV), see Figure S4 in the Supporting Information) is around 25%, corresponding to a Au concentration of around 25 at.%. Therefore, despite their similar Au content, the low Au concentration sample and the composite target sample show totally different distributions within the nanoparticles.

#### 2.1.2 Benchmarking of Atomistic Simulation Model: Global Optimization Analysis

Consequently, the question that needs to be answered is what makes magnetron‐sputtering inert gas condensation from independent Fe and Au targets lead to these distinct vertex/edge‐decorated Fe–Au nanocube morphologies, unlike that from a composite target. As an initial step, nanoparticle shape and phase separation were investigated from a theoretical perspective relying on global optimization calculations.^33^ For this purpose, we developed a potential for atomistic interactions of the Fe–Au system within the second‐moment approximation to the tight‐binding model, often denoted as Gupta potentials (see Section 2 in the Supporting Information for details on the theoretical methodology and the parameters of the interaction potential). The presented Gupta potential was further benchmarked by MD simulations comparing the melting points of Fe and Au nanoparticles, as shown in Figure S5 in the Supporting Information. Results for global optimization calculations of pure Fe nanoparticle shapes are shown in Figure S6a (Supporting Information) for two exemplary sizes (i.e., containing 59 and 169 atoms): truncated rhombic dodecahedral shapes are favored for larger clusters, as in ref. 27 utilizing an embedded‐atom type potential. Optimization of chemical ordering was carried out for mixed nanoclusters by running exchange‐only simulations at fixed geometry (apart from local relaxations).^34^ Figure 1d shows two exemplary Fe‐rich rhombic dodecahedral clusters, namely, Fe_641_Au_30_ and Fe_540_Au_131_, where phase separation is evident. For the low Au concentration case (Fe_641_Au_30_), Au atoms clearly decorate the vertices, whereas for the relatively higher Au concentration case (Fe_540_Au_131_), Au atoms are present both at the vertices and the edges of the nanocluster. Au‐rich cluster compositions go beyond our experimental study, and, hence, the configurational optimization for a Au‐rich case (Fe_100_Au_1189_) is shown in Figure S6b in the Supporting Information. These computational findings are typical for immiscible material systems at the nanoscale as previously reported, e.g., for Au–Co nanoparticles.^35^

It should be stressed that the global optimization calculations show similarities, i.e., the appearance of phase separation, but also important dissimilarities to our growth results. The experimentally observed cuboid morphologies differ from the thermodynamically favorable rhombic dodecahedral shapes and are, hence, a result of kinetic effects. For single‐component Fe nanoparticles synthesized by magnetron‐sputtering inert gas condensation using a single 2'' Fe target, cubic shapes are favored due to competing atomic deposition and surface diffusion mechanisms for a certain range of temperatures and plasma densities.^27^ For the case of Fe–Au nanoparticles presented here, it is anticipated that similar mechanisms govern nanocube formation; however, the influence of co‐sputtering and the incorporation of Au atoms require further clarification.

#### 2.1.3 Synthesis and Characterization of High Au Concentration Sample

**Figure 2:**
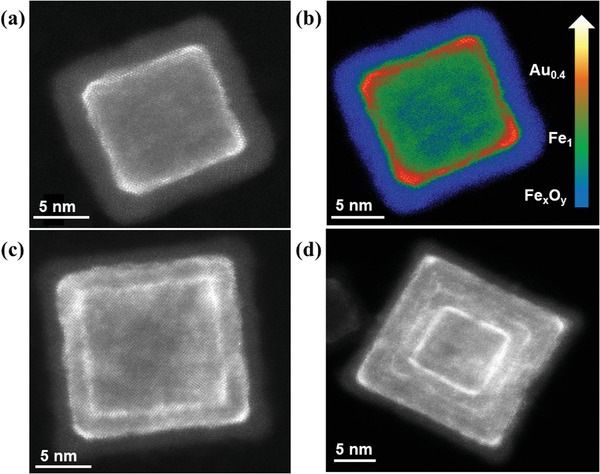
High‐angle annular dark‐field images of Fe–Au nanocubes with increased Au concentration. a) Vertex/edge‐decoration, b) qualitative false color image of the same nanoparticle as in (a) that show the Fe oxide (Fe*_x_*O*_y_* in blue) shell, the Fe (Fe_1_ in green) core, and the Au‐decorated vertices and edges (Au_0.4_ in orange/yellow, 40 at.% Au obtained by EELS quantification), c) one embedded frame‐like Au structure, d) multiple embedded frame‐like Au structures.

Therefore, as a next step, we analyzed the effect of varying synthesis conditions to shed light on the Fe–Au nanocube growth process. When operating the two Fe targets—but not the Au target—during magnetron‐sputtering inert gas condensation (see Table S1 in the Supporting Information), Fe nanoparticles with sizes and morphologies similar to those of the single‐target case discussed above were obtained. Moreover, the size distribution of the Fe nanocube sample (see Figure S2 in the Supporting Information) is almost identical to that of the low Au concentration Fe–Au sample. We infer that co‐sputtering two 1'' Fe targets leads to suitable atomic densities for high deposition rates onto preformed clusters, a prerequisite for the cubic growth regime,^27^ whereas co‐sputtering Au at low concentrations has little influence on the overall nanoparticle size and shape distribution. When adjusting the synthesis conditions for increased Au concentrations (i.e., high Au concentration sample), drastic differences in the morphologies of Fe–Au nanoparticles were observed though. On the one hand, a broadening of the nanoparticle size distribution is accompanied by a decreased probability of cuboid morphologies (see Figure S2 in the Supporting Information). On the other hand, the different gas‐phase growth environment gives rise to Fe–Au nanocubes with complex morphologies. Apart from vertex/edge‐decorated cubes similar to those observed in the low Au concentration sample (see **Figure** **2**a,b), Fe–Au nanocubes with one (Figure 2c) or multiple (Figure 2d) embedded frame‐like structures can be observed. The Au content in this sample is revealed by EELS measurements using the same protocol used for the low Au content sample. A clear increase in Au content and more pronounced vertex‐ and edge‐decoration can be seen from the HAADF‐STEM image in Figure 2b. Although it was anticipated that these surprising morphologies arose from nonequilibrium growth dynamics, further analysis was required to understand the subtle interplay of thermodynamics and kinetic effects.

#### 2.1.4 Growth Mechanism Decomposition by Atomistic Simulation

**Figure 3:**
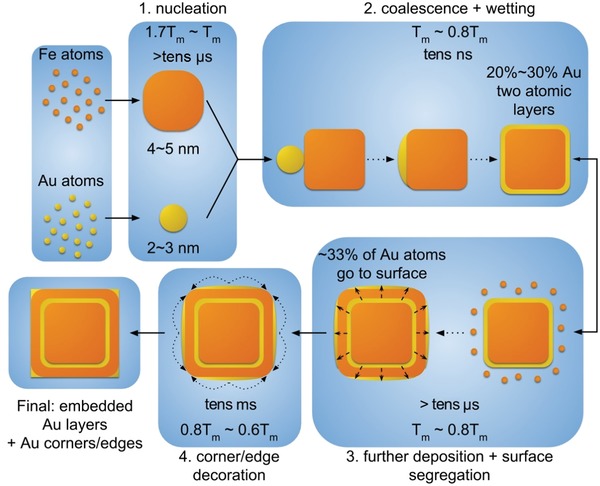
Overview of the multistep Fe–Au nanocube growth model. Nucleation of monometallic clusters (1) is followed by their coalescence and wetting (2). Subsequently, concurrent further deposition of residual Fe atoms in vapor phase and surface segregation of Au atoms in the nanocube create the core‐frame morphology (3). Surface diffusion of Au atoms leads to vertex and edge decoration (4), and eventually to the final multiple‐frame morphology. Relevant time scales and temperatures are indicated. The lower timescale limit of the steps (1) and (3) is estimated from the temperature evolution model^27^ with the fastest cooling rate. The timescale of the step (2) is obtained from the MD simulations shown in Figure 4. The timescale of the step (4) is estimated from the surface migration rate of Au atoms at the given temperatures.

It is known that the morphology of multicomponent nanoparticles obtained by gas‐phase synthesis is governed by nucleation kinetics, coalescence, or deposition processes,^36^, ^37^ as well as complex alloying/segregation characteristics at the nanoscale.^38^, ^39^, ^40^ Clearly, a comprehensive computational model across multiple length and timescales was required to reflect the details of Fe–Au nanocube growth. Consequently, we decomposed the growth process into a series of individual elementary steps corresponding to different driving mechanisms. MD as well as MD combined with Metropolis Monte Carlo (MD + MMC) simulations were employed to study the different stages of the Fe–Au nanocube formation: i) the initial separate nucleation of Fe and Au nanoparticles; ii) the coalescence of preformed Fe and Au nanoparticles; iii) the evolution of Fe–Au nanocubes during further growth due to Fe atom deposition; and iv) the vertex/edge‐decoration with Au atoms due to surface segregation. This multistep mechanism for the growth of Fe–Au nanocubes with embedded Au frame‐like structure is summarized in **Figure** **3**, considering an Fe nanocube with embedded frame‐like Au structure as complex model system to benchmark our simulation approach. Relevant temperatures and timescales are provided in the Figure, whereas individual characteristics of each step of the proposed mechanism are discussed below. Primary condensation of nanoclusters is expected to occur independently for Fe and Au, due to the experimental configuration used which relies on magnetron‐sputtering from independent targets. The relative nucleation and growth rates were assessed by MD simulations of Fe or Au atoms (25 at.%; initial average temperature 900 K) in an environment of Ar atoms (75 at.%; initial average temperature 300 K). Further details on the calculation parameters can be found in Section 2.2 of the Supporting Information. Considering the temporal evolution of the largest cluster in the simulation framework, it was found that the Fe cluster growth rate was approximately twice as high compared with that of Au clusters. Also, after an initial temperature upsurge due to bond formations, both Fe and Au nanoclusters were cooled down to temperatures around their bulk melting points (T_m,bulk_ (Fe) = 1460 K and T_m,bulk_ (Au) = 903 K) within ≈700 ns, due to collisions with Ar atoms. Note that these temperature values are dependent on the metal atom density in the plasma^41^ and that the MD model does not consider cooling due to black‐body radiation, which happens at a much coarser timescale. The marked difference in growth rates is mainly attributed to the large disparity in atomic mass (Fe ∼56 a.u.; Au∼197 a.u.), which results in thermal velocities of Fe atoms being roughly twice as high compared with those of Au atoms. These higher thermal velocities contribute to faster growth rates due to increased collision rates. Further influencing factors would be the stability of dimers and trimers, as well as the plasma charge state, which goes beyond the scope of our computational model. As an example, it was recently shown that Au has a very low nucleation rate,^42^ due to its electronic structure, which forces it to linger in dimeric configurations for much longer than Fe, delaying Au nascent cluster growth significantly, as can also be verified by Figure S7 in the Supporting Information. Taking into account the simulated growth rates and estimations of plasma densities from experimental parameters (see Section 1.1 of the Supporting Information), it can be assumed that the initial condensation process leads to Fe clusters consisting of considerably larger number of atoms compared to Au clusters.

**Figure 4:**
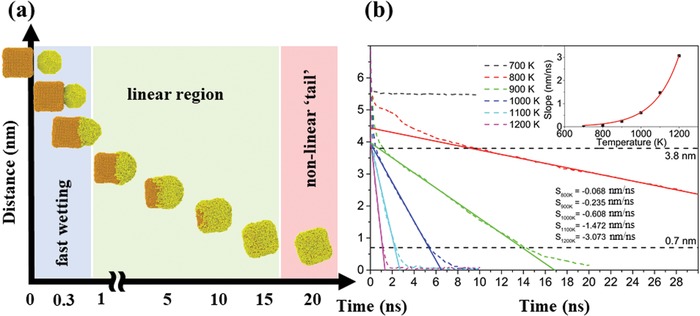
Coalescence behavior of a preformed Fe nanocube with an Au nanocluster. a) MD simulation of the temporal evolution of Fe and Au nanocluster coalescence at initial particle temperatures of 900 K. b) The center of mass distances of Fe and Au during the coalescence process evaluated for MD simulations at different temperatures.

The subsequent coalescence of preformed Fe and Au clusters is considered crucial for the final nanoparticle morphology and was, thus, scrutinized by MD simulations. The temporal evolution of the coalescence event (see **Figure** **4**a) shows that Au atoms rapidly diffuse across the Fe nanocube surface. Note that for the presented conditions with nanocluster temperatures of 900 K the Fe particle is in the solid state, whereas the Au particle is in the liquid phase. In order to quantitatively evaluate the coalescence at different temperatures, the distances between the centers of mass of the Fe and Au nanoclusters were compared (see Figure 4b). It was found that the centers of mass approached each other linearly with time throughout the coalescence process with the slope being exponentially dependent on temperature (inset of Figure 4b). The linear dependence was restricted to center of mass distances between 3.8 and 0.7 nm. Minor deviations from the linear behavior are attributed to fast initial approaching of the clusters followed by wetting (in the beginning) and the diffusion of Au atoms along the cube facet opposite the original interface (forming the tail at the end of the coalescence process). From the MD simulation results we conclude that the temporal evolution during particle coalescence is strongly dependent on the experimental synthesis parameters due to the strong temperature influence. Furthermore, it can be assumed that the coalescence process is finished within a timeframe well below 100 ns for the growth conditions used.

**Figure 5:**
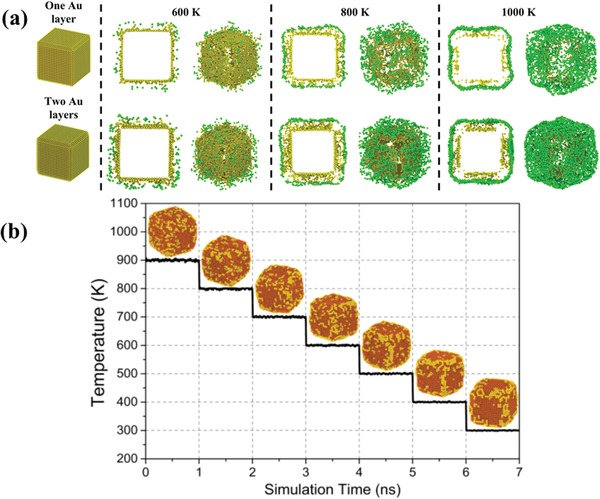
Effect of further deposition of Fe atoms onto the Fe–Au nanocube and subsequent surface segregation. a) MD simulations of nanoparticle growth due to Fe atom deposition onto Fe nanocubes covered with one or two monolayers of Au. The Au atoms were partly embedded by Fe during further growth, but also tended to segregate to the nanocube surface. Embedded and surface gold atoms are depicted yellow and green, respectively. b) Combined MD and MMC simulations demonstrate the vertex/edge‐ decoration of Fe nanocubes with Au atoms due to surface energy minimization.

Keeping in mind that monometallic cluster coalescence occurs inside a supersaturated Fe gas environment, we studied the subsequent growth of the resultant Fe–Au nanocubes owing to further Fe atom deposition. Growth was modeled by placing an Fe nanocube (side length 6.3 nm) covered with one or two monolayers of Au in a MD simulation cell, and adding a new Fe atom every 100fs (see Section 2.2 of the Supporting Information for further details). Results obtained for different temperatures can be seen in **Figure** **5**a: the final configurations of the Fe–Au nanocubes reveal the presence of Au atoms both embedded in an Fe matrix, due to the additional deposition of Fe atoms, and on the surface of the cubes, as a result of energetically driven surface segregation of Au atoms initially found within the cubes. Each particular configuration depends on the competing rates of deposition and segregation, also dictated by the temperature.

Finally, it needs to be clarified how the newly formed Fe–Au nanocubes with surfaces partly covered with Au atoms develop during further cooling. As the simulation of surface diffusion at low temperatures is not feasible by MD due to the prohibitively long‐time scales, a combined MD + MMC method was employed to study the evolution of Au atom configurations on the Fe–Au nanocube surfaces with decreasing temperature. The results shown in Figure 5b demonstrate that initial Au atom decoration of the surfaces is random due to entropy effects, since it happens at high temperatures. However, decoration of cube vertices and edges becomes dominant as the Fe–Au nanocube is approaching room temperature. The driving force behind these configurations can be explained by minimization of surface energy. The Au atoms tend to segregate onto Fe(110) facets to form a Au(111)/Fe(110) interface, as the surface energy of the Au(111) facet (29.9 meV Å^−2^) is lower than that of the Au(100) facet (35.1 meV Å^−2^). However, the differences of interfacial energies are comparatively small (≈0.03 eV per atom) and hence vertex/edge‐decoration with Au atoms becomes insignificant at higher temperatures. Note that the proposed Au(111)/Fe(110) interfaces are in excellent agreement with experimental observations from high resolution transmission electron microscopy (see Figure S8 in the Supporting Information).

Hence, our multistage simulation approach was successful in reproducing the experimentally observed Fe nanocubes with one embedded frame‐like Au structure. To explain the other Fe–Au nanocube morphologies presented above, one has to consider different size ratios of the coalescing Fe nanocubes and Au clusters, coalescence/wetting happening at different growth stages (i.e., at varying distances from the magnetron sputter target) and multiple coalescence events. It has to be noted that Fe–Au nanoparticles with cuboid Fe cores and epitaxial interfaces can also be ascribed to thermodynamics considerations, as demonstrated in ref. 20. Indeed, the presented Gupta potential was able to reproduce epitaxial Au (100)/Fe (100) interfaces in MD + MMC calculations, which were aimed at studying the effects of annealing on randomly mixed alloy configurations (see Figure S9 in the Supporting Information). Nevertheless, due to the compelling agreement between experimental results and MD modeling, we attribute the complex morphologies of Fe–Au nanocubes to kinetic effects, which were further corroborated by in situ heating experiments inside the transmission electron microscope. The embedded Au frame of a Fe–Au nanocube was not stable upon heating, which resulted in Au atoms diffusing to the Fe/Fe oxide interface (see Figure S10 in Supporting Information); hence, we conclude that the presented Fe–Au nanoparticles show metastable characteristics rather than being equilibrium structures.

## 3 Conclusion

In summary, we experimentally demonstrated the gas‐phase synthesis of phase‐separated Fe–Au nanocubes with complex morphologies by a magnetron‐sputtering inert gas condensation approach. These hybrid nanoparticles are promising candidates for future developments toward applications in, for instance, catalysis,^43^ as well as biomedicine, e.g., for in vivo drug delivery^44^ or as multimodal contrast agents in magnetic resonance imaging.^45^ Furthermore, we elaborated a detailed multistep model for the growth of Fe–Au nanocubes, which can serve as general framework with guiding significance for the synthesis of hybrid nanoparticles, both from an experimental and theoretical perspective. Considering immiscible material systems similar to Fe–Au, it is expected that the proposed growth model is widely applicable for the gas‐phase synthesis of hybrid nanoparticles from independent source targets. Also, it will be an interesting future research direction to study the differences for materials systems presenting miscibility gaps and similar melting points, for instance alloy Fe–Pd nanocubes that have been recently demonstrated using a similar experimental approach.^46^ In addition, the present work includes the development, benchmarking, and application of an optimized atomistic potential for interactions of Fe and Au, which will stimulate future works on this system ranging from nanoalloys to bulk materials. Finally, the presented theoretical framework can be easily adapted for a multitude of material systems, which will give important insights on the interplay between thermodynamics and kinetics during the growth of hybrid nanoparticles.

## 4 Experimental Section

*Sample Preparation*: The Fe and FeAu nanoparticles were prepared using a cluster beam deposition (CBD) technique based on the magnetron‐sputtering process. A commercial Nanogen Trio (Mantis Ltd) nanoparticle source (see Figure S1, Supporting Information) was used, which consisted of three individual targets placed in‐plane at equal distance to each other (aggregation length fixed at 95 mm). In the experiments, a supersaturated vapor of metal atoms was produced by co‐sputtering of three individual targets (two Fe and one Au) in an argon (Ar) atmosphere. The aggregation zone was water‐cooled and pumped down to ≈10^−6^ mbar, prior to sputtering. High purity Fe (1” diameter, 99.9%) and Au (1” diameter, 99.995%) targets were used in the DC co‐sputtering process.

*Physical and Chemical Characterization*: Synthesized nanoparticles were analyzed using an image‐corrected scanning/transmission electron microscope (S/TEM) FEI Titan 80–300 kV operated at 300 kV. The crystalline structure of the nanoparticles was determined by high‐resolution TEM (HRTEM) analysis. The identification of the nanoparticle morphology (Fe–Au core‐frame) was obtained by the Z‐contrast of HAADF‐STEM imaging (Z is the elemental atomic number). Electron energy loss spectroscopy was performed to study the Fe and Au distributions within the nanoparticles using a Gatan GIF Quantum energy filter, as well as to estimate qualitatively the Au content within the nanoparticle at different positions. The chemical composition and oxidation state were also evaluated by X‐ray photoelectron spectroscopy , using a Kratos Axis UltraDLD 39–306 equipped with a monochromated Al Kα source operated at 300 W. The binding energy scale was calibrated by measuring the C1s peak at 284.8 eV, and the binding energy of Au 4f and Fe 2p was investigated.

*Computational Simulation Methods*: All atomistic simulations were performed with the classical MD code LAMMPS. The interactions between Au–Au, Au–Fe, and Fe–Fe were modeled with the Gupta potential described in the Supplementary Information, where additional details for every simulation step can also be found.

## Conflict of Interest

The authors declare no conflict of interest.

## Supporting information

Supplementary
