# Supplementary material for: Site‐Specific Wetting of Iron Nanocubes by Gold Atoms in Gas‐Phase Synthesis
Source: Adv Sci (Weinh). 2019 May 2;6(13):1900447. doi: 10.1002/advs.201900447 (PMC6662390; doi:10.1002/advs.201900447)
Supplement: Supplementary file 1 — Supplementary [file ADVS-6-1900447-s001.pdf]

Copyright WILEY-VCH Verlag GmbH & Co. KGaA, 69469 Weinheim, Germany, 2019.

ADVANCED SCIENCE
Open Access

Supporting Information

for *Adv. Sci.,* DOI: 10.1002/advs.201900447

Site-Specific Wetting of Iron Nanocubes by Gold Atoms in Gas-Phase Synthesis

Jerome Vernieres, Stephan Steinhauer, Junlei Zhao,* Panagiotis Grammatikopoulos, Riccardo Ferrando, Kai Nordlund, Flyura Djurabekova, and Mukhles Sowwan*

Copyright WILEY-VCH Verlag GmbH & Co. KGaA, 69469 Weinheim, Germany, 2019.

Supporting Information

Site-specific wetting of iron nanocubes by gold atoms in gas-phase synthesis

Jerome Vernieres, Stephan Steinhauer, Junlei Zhao*, Panagiotis Grammatikopoulos, Riccardo Ferrando, Kai Nordlund, Flyura Djurabekova, Mukhles Sowwan*

Contents

1 Supplemental data

1.1 Materials and methods

1.2 Additional TEM images of each samples with their respective size distribution

1.3 Electron Energy Loss Spectroscopy (EELS) quantification method

1.4 Additional X-Ray Photoelectron Spectroscopy (XPS) data

1.5 Dependence of the melting points on cluster size and potential

1.6 Optimization of physical and chemical ordering of Fe and Fe-Au clusters

1.7 Lifetime simulation of dimer and trimer for both elements (Fe and Au)

1.8 HRTEM analysis of the Fe/Au interface

1.9 MD + MMC simulation of the Au-Fe interface

1.10 Stability of the Au embedded layer upon heating

2 Theoretical methodology

2.1 Fe-Au potential optimization

2.1.1 The Gupta potential

2.1.2 Au-Au interactions

2.1.3 Fe-Fe interactions

2.1.4 Fe-Au interactions

2.2 Computational simulation methods

3 References

1 Supplemental data

1.1 Materials and methods

In this study, three different samples were investigated: (i) a low-Au concentration sample (Table S1); (ii) a pure Fe sample (Table S2) and (iii) a high-Au concentration sample (Table S3).

Table S1. Experimental parameters of the low-Au concentration sample

Materials	V_{dc} [V]	E [eV]	Y^{AR}	ϕ_{AR} [sccm]	P_{AR} [bar]	ρ [m^{-3}]
Au	262	192	0.76	72	2.5×10^{-4}	1.2×10^{19}
Fe_1	230	168	0.42	72	2.5×10^{-4}	4.3×10^{-19}
Fe_2	230	168	0.42	72	2.5×10^{-4}	4.3×10^{-19}

Table S2. Experimental parameters of the pure Fe sample

Materials	V_{dc} [V]	E [eV]	Y^{AR}	ϕ_{AR} [sccm]	P_{AR} [bar]	ρ [m^{-3}]
Fe_1	263	193	0.49	58	2×10^{-4}	4.8×10^{-19}
Fe_2	264	193	0.49	58	2×10^{-4}	4.8×10^{-19}

Table S3. Experimental parameters of the high-Au concentration sample

Materials	V_{dc} [V]	E [eV]	Y^{AR}	ϕ_{AR} [sccm]	P_{AR} [bar]	ρ [m^{-3}]
Au	271	199	0.79	64	2×10^{-4}	1.4×10^{19}
Fe_1	246	180	0.46	64	2×10^{-4}	3.8×10^{-19}
Fe_2	246	180	0.46	64	2×10^{-4}	3.8×10^{-19}

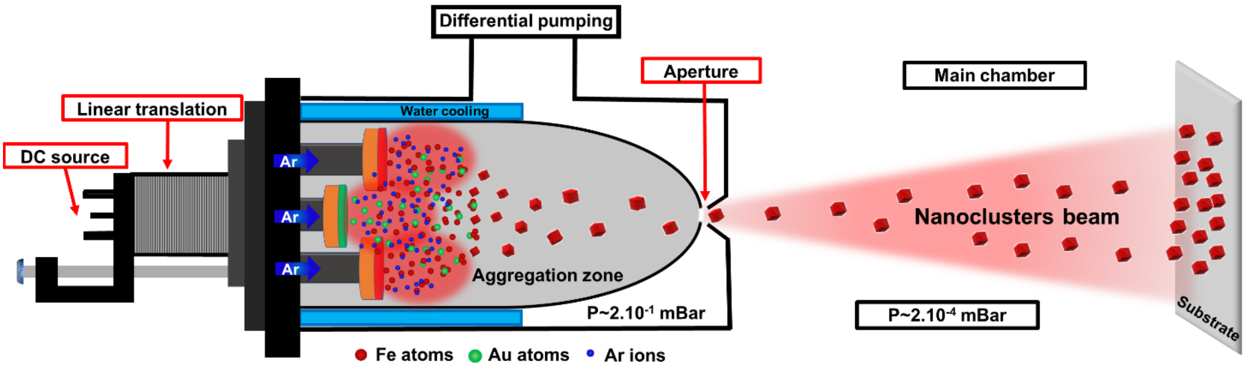

Figure S1. Schematic illustration of the magnetron-sputtering inert gas condensation system used to produce the FeAu nanocubes.

1.2 Additional TEM images of each samples with their respective size distribution

The size distribution of each sample has been deduced from more than 200 nanoparticles in all cases. Figure S2b, Figure S2d and Figure S2f show the size distribution of the three different samples. In each size distribution, the shapes of the nanoparticles are considered, with the abbreviations "NPs" and NCs" corresponding to spherical nanoparticles and cuboid nanoparticles, respectively.

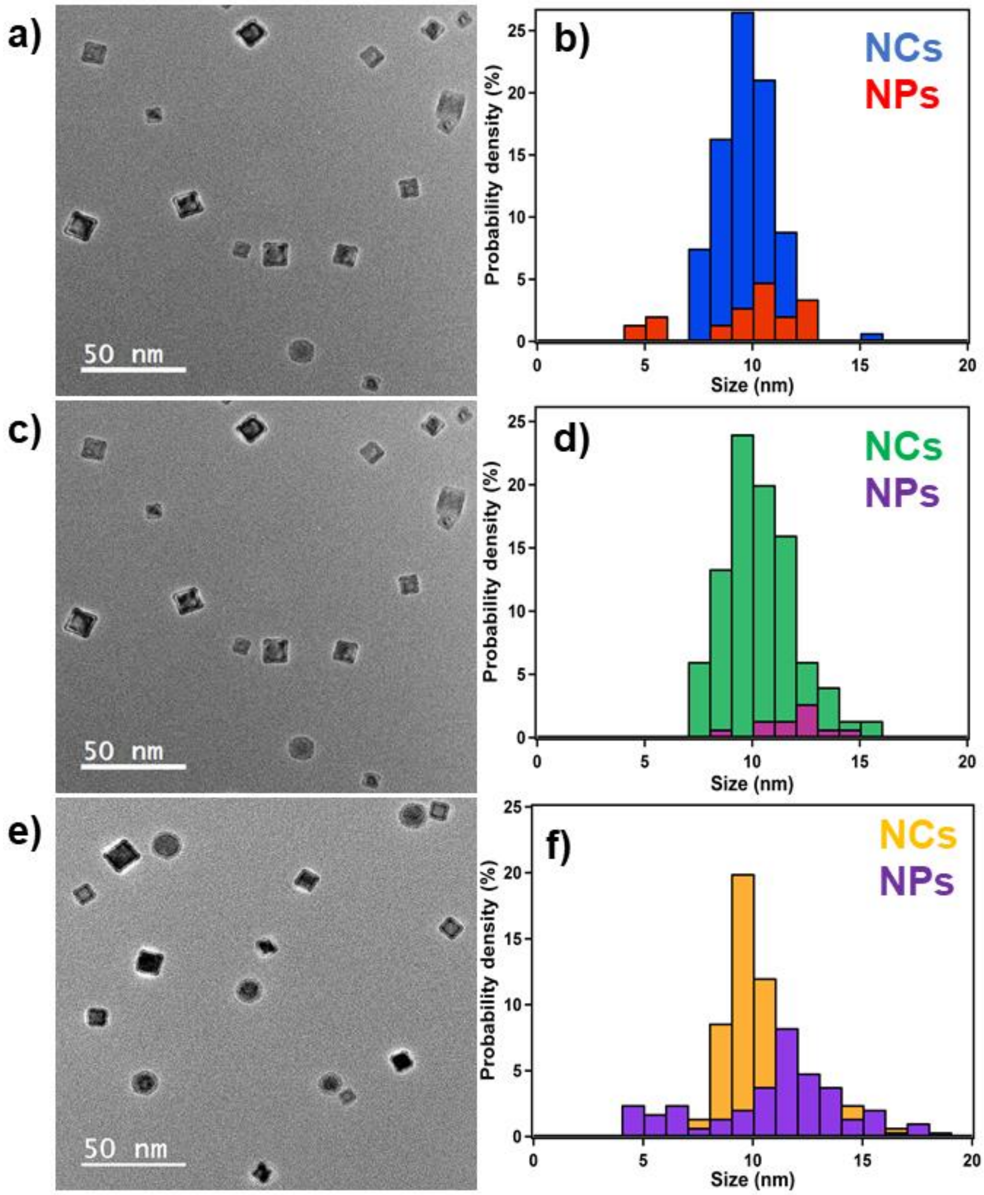

Figure S2. Transmission electron microscopy and corresponding size distribution of the as-deposited nanoparticles after air exposure. a) Overview TEM images of FeAu sample with low-Au concentration and b) corresponding size distribution c) Overview TEM images of pure Fe sample and d) corresponding size distribution e) Overview TEM images of FeAu sample with high-Au concentration and f) corresponding size distribution.

1.3 Electron Energy Loss Spectroscopy (EELS) quantification method

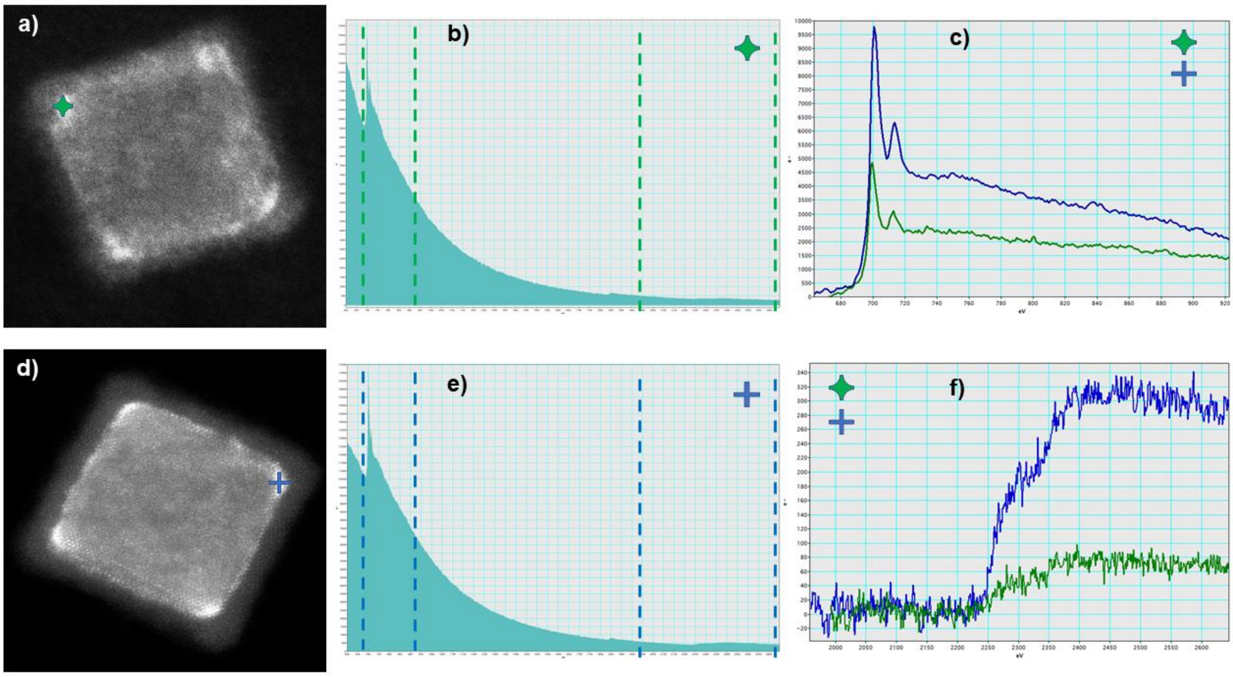

Figure S3. EELS point-scan quantification using Digital Micrograph software. a) and d) show STEM images of a representative FeAu nanocube from the low-Au concentration sample and from the high-Au concentration sample, respectively. b) and e) are the corresponding EELS spectra acquired on the green marker shown in (a) and blue marker shown in (d). c) and f) Comparison of the background-subtracted Fe spectra and Au spectra for both point scans.

1.4 Additional X-Ray Photoelectron Spectroscopy (XPS) data

Elemental characterization of the as-prepared Fe-Au nanoparticles was performed after exposure to air, using X-Ray Photoelectron Spectroscopy (XPS). The analysis chamber base pressure was 1.6×10^{-9} mbar. The X-ray source used was a monochromatized Al Kα (1486.6 eV). The employed pass energy was 160 eV and 20 eV for the wide scan and high-resolution spectra, respectively. The atomic concentrations were quantified by integration of the Au 4f and Fe 2p peaks after background subtraction. A Shirley-type background was chosen for all XPS spectra. The intensities were corrected using the corresponding relative sensitivity factors for each element. The XPS spectra fitting was performed with a combination of Gaussian/Lorentzian product form (GL) and Gaussian/Lorentzian Sum form (SGL), as line-shapes, using CASA XPS software.

Carbon was fitted using four different features, which we attribute to carbon and its surface oxides. Oxygen was fitted using three different features corresponding to the oxygen in SiO_2, the metal Hydroxide (OH) and the metal oxide contributions. Au was fitted using doublet function for the metallic state. Fe was fitted using one peak for the metallic state (~706 eV), two peaks for the iron oxide and another couple of peaks for the satellite features.

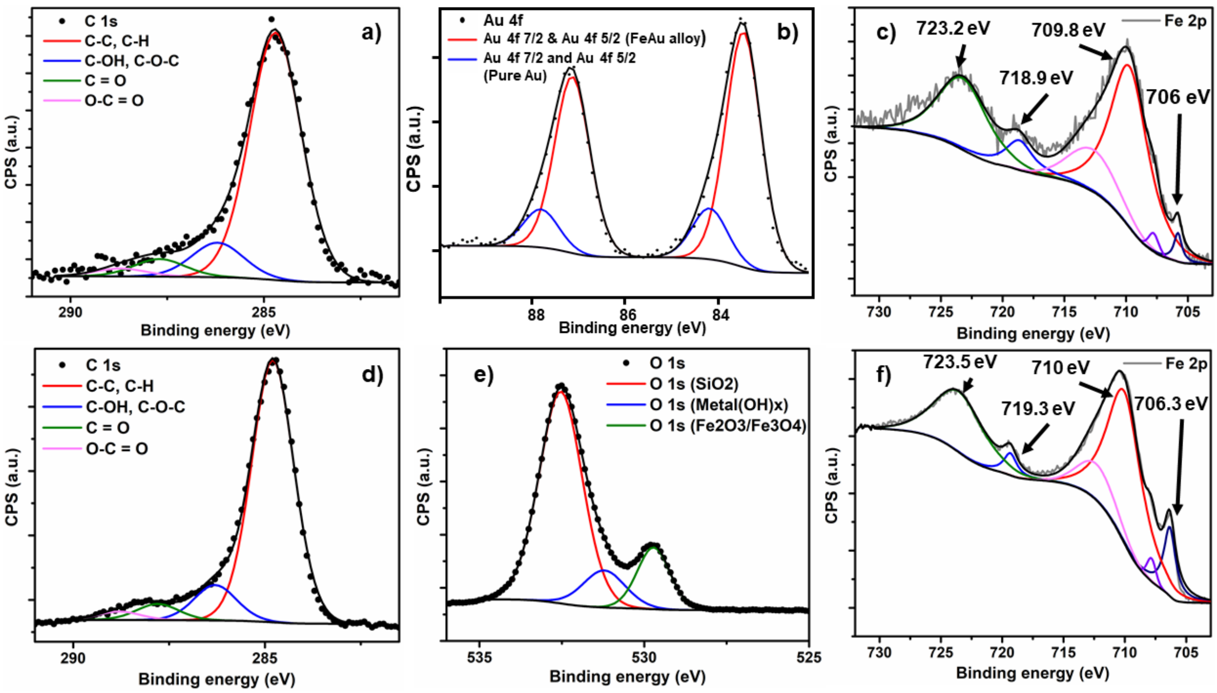

Figure S4. High-resolution XPS spectra. a) to c) FeAu alloy nanocube sample grown using the composite target. d) to f) Phase-separated Fe-Au nanocube sample grown by co-sputtering with low-Au concentration.

1.5 Dependence of the melting points on cluster size and potential

In order to understand the relative state of Au and Fe NPs before coalescence, we run benchmarking simulations on the size dependence of the melting point of pure Au and Fe NPs. The simulations were performed by heating up the solid nanoparticle from room temperature to the melting point and beyond at a heat rate of 200 K/ns. The initial shape of the nanoparticles was derived from the Wulff construction.[1] The applied thermostat was the Nosé-Hoover thermostat.[2,3] Linear and angular momenta were removed for the nanoparticles. We compared the results using the present Gupta potential with those for established and commonly used Embedded-Atom-Method (EAM) potentials.[4,5] The method is known to produce slightly higher melting points because of the relatively high heat rate; therefore, we compared the obtained curves with the coexisting liquid-solid phase simulations[6] on corresponding bulk materials.

As shown in Figure S5, the Gupta potential used in this work gives lower melting points of both Au and Fe nanoparticles. However, the relative tendency is very similar to the reference potentials (Mendelev et al.[5] for Fe and Foiles et al.[4,7] for Au). The results show that the melting point of the intermediate-size Fe nanoparticle (N ≥ 2000) is already higher than the bulk melting point of Au ($T_{Au}^{Exp} = 1337\,K$), indicating that, during coalescence, Au nanoparticles are most probably in liquid state, while Fe nanoparticles at the same temperature are solid.

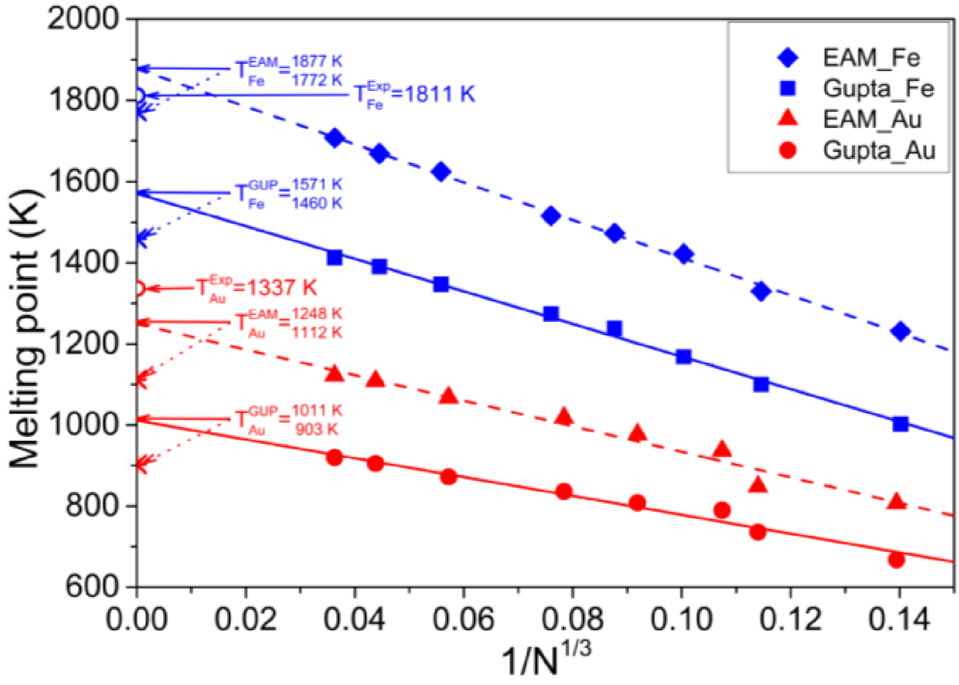

Figure S5. Dependence of melting points on cluster size and potential. The melting points of pure Au and Fe nanoparticles are linearly fitted to a function of $N^{-1/3}$, respectively. Two reference potentials are used to compare with our Gupta potential. Au is compared to extrapolation of linear fitting to bulk ($N^{-1/3} = 0$) is a bit higher than the values obtained from coexisting liquid-solid phases simulations.[6]

1.6 Optimization of chemical ordering for Fe-Au clusters

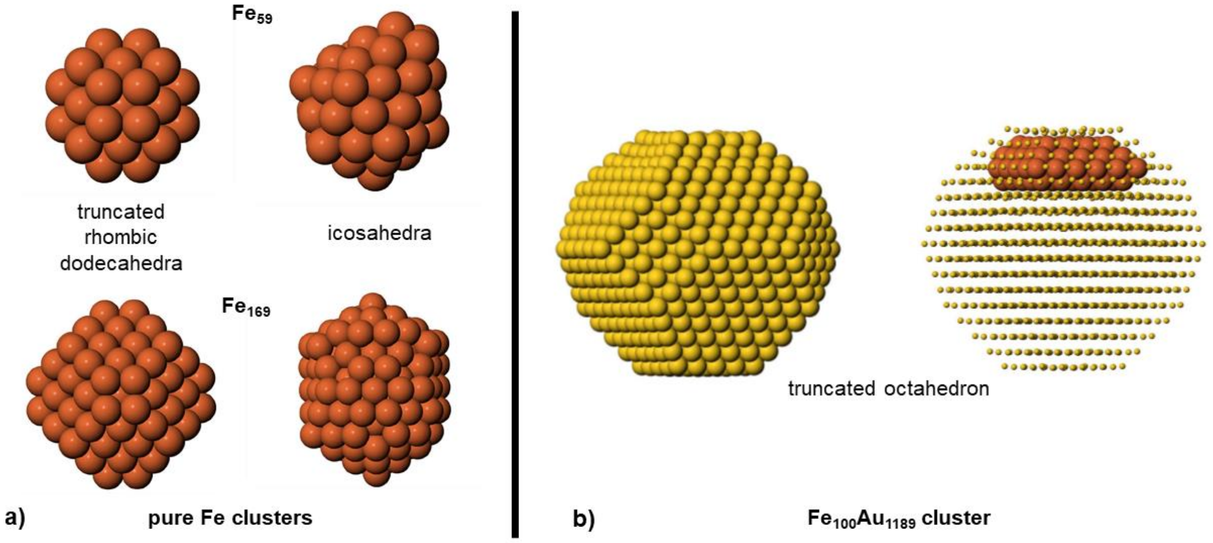

Figure S6. Atomistic global optimization simulations: a) Physical ordering of pure Fe clusters containing 59 and 169 atoms. For the smaller size, the icosahedral structure was more stable by ~1.2 eV, whereas for the larger size, the truncated rhombic dodecahedral shape was slightly more favorable by ~0.1 eV. b) Chemical ordering of a Au-rich cluster with an fcc, truncated octahedron structure, showing elemental segregation, with an off-center Fe bcc inner core optimally located at a subsurface position.

1.7 Lifetime simulation of dimer and trimer for both elements (Fe and Au)

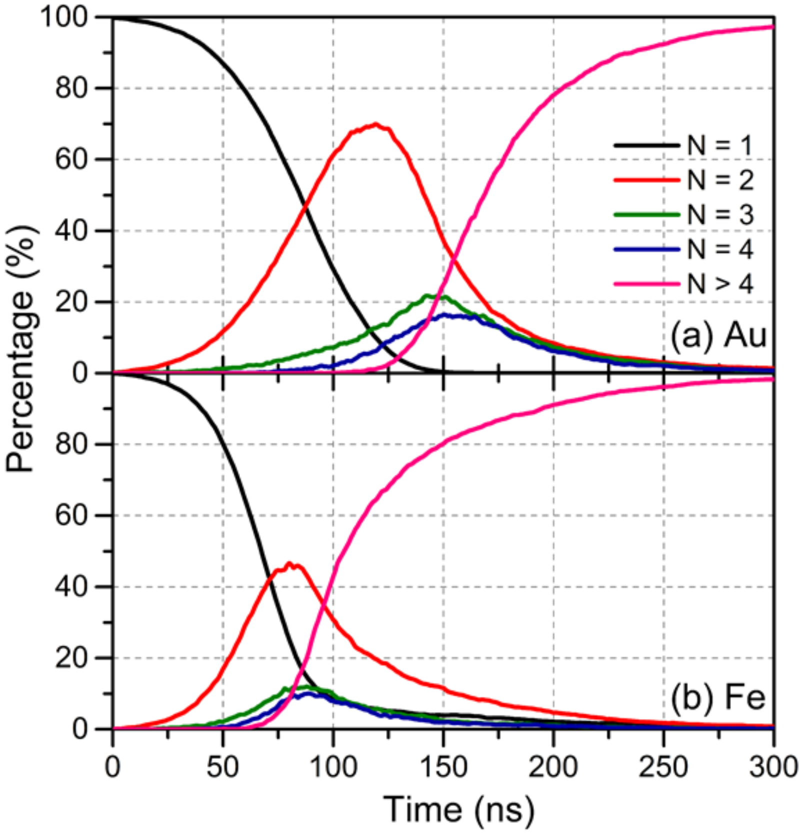

Figure S7. Percentage of atoms of each species bound in monomers (N=1), dimers (N=2), trimers (N=3) and tetramers (N=4), etc. as a function of time during MD simulation of gas condensation. Clearly, Au dimers reach much higher percentages and have much longer lifetimes compared with Fe dimers.

1.8 HRTEM analysis of the Fe/Au interface

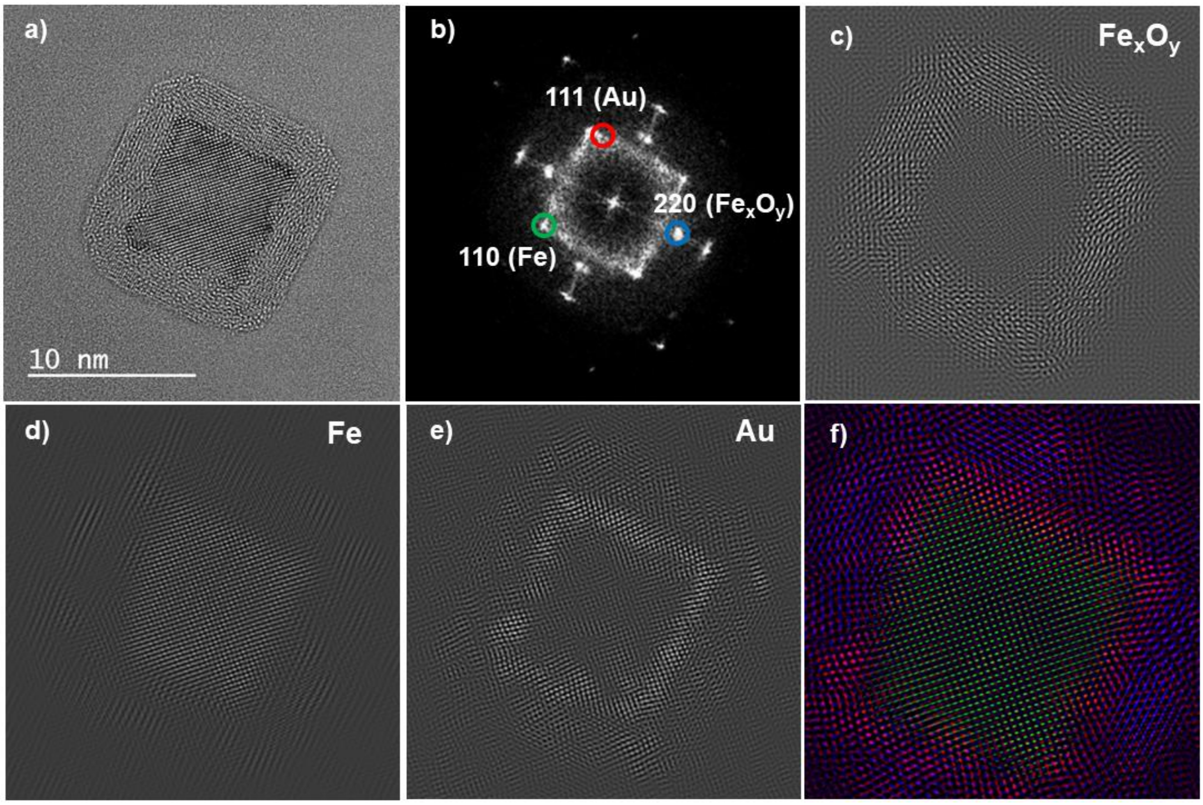

Figure S8. a) Representative HRTEM image of an FeAu nanocube from the low-Au concentration sample. A clear core-shell morphology is revealed with a highly crystalline core. b) Corresponding FFT pattern of the whole HRTEM image. From this FFT, we identified spots attributed to the Fe metallic phase (bcc α-Fe), to the Fe oxide phase (γ-Fe_2O_3 and/or Fe_3O_4), as well as to the Au phase (fcc). c), d) and e) represent each elemental crystalline phase within the nanocube. We can clearly observe the Fe oxide (c) surrounding the Fe metallic core (d). Interestingly, we can also observe that the Au is located at the interface between the core and shell of the nanocube (e). f) Finally, we merged all the masked images in a single one (enlarged and color-coded) in order to visualize the distribution of each element within the nanocube more clearly.

1.9 MD + KMC simulation of the Au-Fe interface

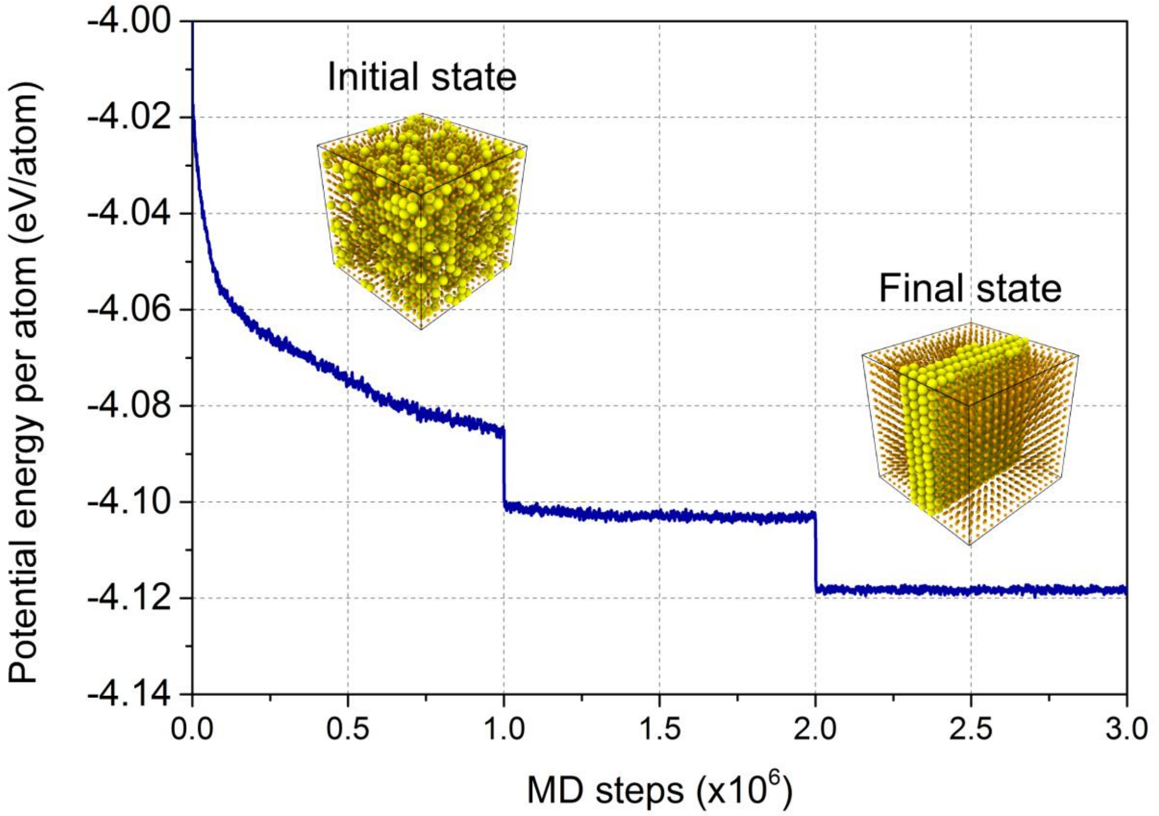

Figure S9. MD+MMC annealing of bulk Au/Fe alloy. Initially, the Au and Fe atoms were randomly mixed. The whole simulations went through 600, 500, and 400 K for 106 MD steps each. The final ground state consists of a layered configuration with a Au(100)/Fe(100) interface.

1.10 Stability of the Au embedded layer upon heating

In-situ heating inside the electron microscope column was achieved with the Protochips Aduro 500 TEM holder platform, relying on membrane-based heating chips operated with open loop temperature control. Annealing experiments in vacuum were conducted at 500°C at pressures in the 10^{-7} mbar range.

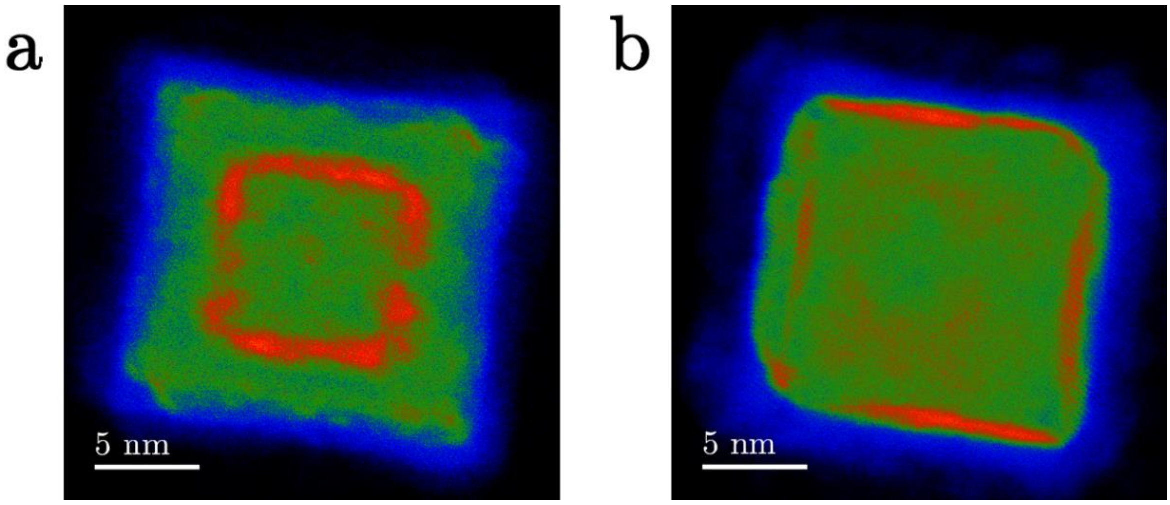

Figure S10. Stability of the Fe-Au naocube morphology and of the embedded Au layer heating in vacuum. Scanning transmission electron microscopy of Fe-Au nanocube with embedded frame-like structure, a) before and b) after in situ annealing at 500°C for 40 min inside the TEM column. The embedded Au atoms diffused to the Fe – Fe oxide interface upon heating, which corroborates the metastable nature of the frame-like structures.

2 Theoretical methodology

2.1 Fe-Au potential optimization

2.1.1 The Gupta potential

The atomistic potential employed in our calculations is derived within the second-moment approximation to the tight-binding model.[8-11] It is often denoted as Gupta potential in the literature. Gupta potential is many-body, because it cannot be written as the sum of pair terms. The potential energy E_{pot} of a cluster of N atoms is written as the sum of single-atom contributions:

$$E_{pot} = \sum_{i}^{N} \left[E_{rep}(i) + E_{attr}(i) \right] \tag{1}$$

$E_{rep}(i)$ and $E_{attr}(i)$ represent repulsive and attractive contributions, respectively, and are defined as

$$E_{rep}(i) = \sum_{i \neq j, r_{ij} \leq r_{c,ab}} A_{ab} \exp\left(-p_{ab}\left(\frac{r_{ij}}{r_{0,ab}} - 1\right)\right) \tag{2}$$

$$E_{attr}(i) = \left[\sum_{i \neq j, r_{ij} \leq r_{c,ab}} \xi_{ab}^{2}\, exp\left(-2q_{ab}\left(\frac{r_{ij}}{r_{0,ab}} - 1\right)\right)\right]^{\frac{1}{2}} \tag{3}$$

Where $a(b)$ represent the atomic species of atom i (j), r_{ij} is the distance between these atoms, and p, q, A, ξ and r_0 are adjustable parameters. Thus, for a binary system a set of 15 parameters (5 for each element plus 5 describing the mixing, because (a, b) and (b, a) parameters are the same) need to be defined, of which only 12 are independent (it is always possible to adjust the other parameters to changes in r_0). r_c is the cut-off radius. Beyond r_c, the potential is smoothly brought to zero at a distance r_{c2} by a fifth order polynomial. The choices of r_c and r_{c2} for the different types of interactions are explained in the following sections. The parameters of the Gupta potential are given in Table S4. In the next sections we comment about their choice.

2.1.2 Au-Au interactions

Au-Au parameters are taken from.[12] They have been tested against experimental data for gas-phase Au clusters[13] and for Au clusters adsorbed on MgO(100)[12-14] obtaining a quite good agreement. r_c and r_{c2} have been chosen as the second-neighbor and third-neighbor distances in Au, respectively.

Table S4. Parameters of the FeAu Gupta potential

Parameter	Au-Au	Fe-Fe	Fe-Au
p	10.139	11.24	10.6895
q	4.033	2.136	3.0845
A (eV)	0.20957	0.119552	0.164561
ξ (eV)	1.8153	1.554741	1.6659
r_0 (Å)	2.885	2.48246	2.68373

2.1.3 Fe-Fe interactions

The four independent parameters of Fe-Fe interactions have been fitted to the equilibrium nearest-neighbor distance in bulk bcc α-iron, to the bulk modulus (170 GPa, see https://www.webelements.com/iron/physics.html), to the cohesive energy of α-iron (4.28 eV/atom, see Ref. 15), and to the difference between fcc and bcc bulk phases extrapolated to $T = 0$, which is 57 meV per atom.[16] Reproducing the correct energy difference between fcc and bcc phases is crucial for stabilizing the latter and obtaining the correct cluster shapes with increasing size. The cutoff radii r_c and r_{c2} have been chosen as the third-neighbour distance in bcc α-iron and the third-neighbor distances in fcc γ-iron, respectively.

This potential gives a vacancy formation energy of 1.46 eV, in excellent agreement with the experimental value of 1.4 ± 0.1 eV.[17] The surface energies γ for some low-index surfaces of Fe are given in Table S4. These values are somewhat smaller then those obtained by DFT,[18] which are in the range of 140 meV/Å^2. However, since Gupta potential underestimates also Au surface energies, the difference in surface energy of the two metals is well reproduced. The difference in surface energies is a key driving forces for surface segregation.

2.1.4 Fe-Au interactions

For Fe-Au interactions, the parameters p, q, A, r_0 have been taken as the arithmetic averages of those of pure metals, while ξ has been fitted to the dissolution energy of a single impurity in bulk Au, for which detailed experimental data are available. This energy is of 0.30 eV/atom.[19] The dissolution energy of Au in Fe has then been calculated, obtaining the much large value 0.67 eV/atom, in qualitative agreement with the experimental results of a weaker miscibility of Au in Fe than Fe in Au. Cutoff distances r_c and r_{c2} are of 4.06692 and 4.712589 Å, respectively.

Table S5. Surface energies (in meV/Å^2) for bcc Fe surfaces

surface	γ
(110)	95.3
(100)	106.3
(111)	113.6

2.2 Computational simulation methods

In this work, the computational simulations we performed can be grouped as follows: (i) The initial nucleation of Au and Fe nanoparticles, (ii) the coalescence of Au and Fe nanoparticles, (iii) the further deposition and surface segregation and (iv) MD combined with Metropolis Monte Carlo (MMC) annealing of the Au surface migration. All the simulations were performed with the classical MD code LAMMPS.[20] Besides the main simulations, we also did the benchmarking simulations on the melting points of pure Au and Fe NPs (see section 1.5 of the Supplementary information). The interactions between Au-Au, Au-Fe and Fe-Fe were modelled with the Gupta potential described in the previous section.

In the simulations of the initial nucleation, we studied the nucleation rates of Au and Fe plasma with the cooling of the Ar atmosphere. The Ar atmosphere thermostat has already been used in previous studies.[21,22] Each simulation consisted of 12,500 Ar atoms and 3,125 pure metallic (Au or Fe) atoms. Initially, the atoms were randomly placed in a cube cell of 100 nm in side length, followed by primary energy minimization to avoid “hot spots” in the system. The initial temperature of Ar atoms was set to 300 K, while the temperature of the metallic atoms was 900 K. The velocities of atoms followed the Gaussian distribution. The total linear and angular momentum of the system were set to zero. The temperature of the Ar

atmosphere was scaled with the Nosé-Hoover thermostat,[3] while the metallic atoms were allowed to evolve freely and be cooled down by collisions with Ar atoms. The whole system can be considered as a NVT ensemble with the initial conditions far from equilibrium. The Ar-Ar interaction was modelled with Lennard-Jones potential.[23] The Ar-Au and Ar-Fe interactions were given by the corresponding purely repulsive Ziegler-Biersack-Littmark (ZBL) potentials.[24] Five cases with different initial atomic positions and velocities were run for 700 ns with timestep of 1fs.

The initial state of the coalescence simulation is shown in Figure 4a. The initial positions of the Au and Fe nanoparticles were set at 10 Å away from each other, in order to avoid the mutual interactions before the thermostatting. The numbers of atoms are 20922 for the cubic Fe nanoparticle and 4033 for the truncated octahedral Au nanoparticle. The initial temperatures for both nanoparticles were set from 700 K to 1200 K (100 K per step) in each case, respectively. After the initial thermalization, the thermostat was removed and two nanoparticles were given a relative drift velocity 10 m/s. The coalescence process was simulated in NVE ensemble (See Supporting Information Movie S1).

Subsequent simulations of further deposition used the approach describe in a previous study.[25] A cubic Fe nanoparticle with a Au outer shell (containing 20922 atoms, 6.3 nm side length), was placed in the center of a 20 nm × 20 nm × 20 nm simulation box. The Nosé-Hoover thermostat was applied to the atoms initially located within the 5 nm spherical region at the center of the nanoparticle. The deposition was simulated by adding a new Fe atom into the cell every 100 MD steps and 30,000 Fe atoms were added in total. We compared the final structure of the grown nanoparticles at 800 and 1000 K with 23.5 % (two-layers shell) and 13.2 % (one-layer shell) Au concentration. The simulation was carried out for 4 ns with a time step of 1 fs (See Supporting Information Movie S2).

Surface decoration is purely a diffusion process, taking place after the deposition process had finished. The time scale of the thermal activated process is beyond the capabilities of MD at low temperature. The conventional solution of increasing the temperature would enhance the entropy effect, thus changing the thermodynamics significantly. Therefore, in order to found the equilibrium configuration of the surface, we conducted simulated annealing using a combined MD + MMC method. The initial structure was an Fe nanocube with 12471 atoms. The 30 % outermost surface atoms (i.e. 576 out of 1976 atoms) were switched to Au randomly. Then, the whole system was simulated in NVT assemble, starting from 900 K to 300 K. During the simulation, the Au and Fe atoms were allowed to swap positions randomly every 10 MD steps. The acceptance rate followed the Metropolis criterion: $P = \exp\left(\frac{-\Delta E}{k_B T}\right)$, for potential energy difference between the $\Delta E \geq 0$; while $P = 1$, if $\Delta E < 0$, where k_B and T are the Boltzmann constant and the corresponding temperature, respectively. The simulations ran 1 ns for each temperature step and the temperature step was set at 100 K (900 K, 800K …, 300K).

3 References

[1] G. Wulff, Z. Kristallogr. *Cryst. Mater.* **1901**, 34, 449.

[2] S. J. Nosé, *Chem. Phys.* **1984**, 81, 511.

[3] W. G. Hoover, *Phys. Rev. A* **1985**, 31, 1695.

[4] S. M. Foiles, M. I. Baskes, M. S. Daw, *Phys. Rev. B* **1986**, 33, 7983, Erratum: ibid, *Phys. Rev. B* **1988**, 37, 10378.

[5] M. I. Mendelev, S. Han, D. J. Srolovitz, G. J. Ackland, D. Y. Sun, M. Asta, *Philos. Mag.* **2003**, 83, 3977.

[6] S. Yoo, X. C. Zeng, J. R. Morris, *J. Chem. Phys.* **2004**, 120, 1654.

[7] S. M. Foiles, J. B. Adams, *Phys. Rev. B* **1989**, 40, 5909.

[8] F. Cyrot-Lackmann, F. Ducastelle, *Phys. Rev. B* **1971**, 4, 2406.

[9] R. P. Gupta, *Phys. Rev. B* **1981**, 23, 6265.

[10] V. Rosato, M. Guillope, B. Legrand, *Phil. Mag. A* **1989**, 59, 321.

[11] F. Cleri, V. Rosato, *Phys. Rev. B* **1993**, 48, 22.

[12] R. Ferrando, G. Rossi, A. C. Levi, Z. Kuntova, F. Nita, G. Barcaro, A. Fortunelli, A. Jelea, C. Mottet, J. Goniakowski, *J. Chem. Phys.* **2009**, 130, 174702.

[13] D. M. Wells, G. Rossi, R. Ferrando, R. E. Palmer, *Nanoscale* **2015**, 7, 6498.

[14] Y. Han, R. Ferrando, Z. Y. Li, *J. Phys. Chem. Lett.* **2014**, 5, 131.

[15] C. Kittel. *Introduction to Solid State Physics*, University of California, Berkeley, **2005**.

[16] L. Kaufman, E. V. Clougherty, R. J. Weiss, *Acta Metallurgica* **1963**, 11, 323.

[17] S. M. Kim, W. J. L. Buyers, *J. Phys. F: Metal Physics* **1978**, 8, 103.

[18] A. Hung, I. Yarovsky, J. Muscat, S. Russo, I. Snook, R. O. Watts, *Surface Science* **2002**, 501, 261.

[19] L. Topor, O. J. Kleppa, *Metallurgical Transactions* **1984**, 15, 573.

[20] S. J. Plimpton, *Comput. Phys.* **1995**, 117, 1.

[21] E. Kesala, A. Kuronen, K. Nordlund, *Phys. Rev. B* **2007**, 75, 174121.

[22] J. Zhao, V. Singh, P. Grammatikopoulos, C. Cassidy, K. Aranishi, M. Sowwan, K. Nordlund, F. Djurabekova, *Phys. Rev. B* **2015**, 91, 035419.

[23] J. E. Jones, *Proceedings of the Royal Society of London*. Series A, Containing Papers of a Mathematical and Physical Character **1924**, 106, 463.

[24] J. F. Ziegler, J. P. Biersack, U. Littmark, *The Stopping and Range of Ions in Matter,* Pergamon, New York, **1985**.

[25] J. Zhao, E. Baibuz, J. Vernieres, P. Grammatikopoulos, V. Jansson, M. Nagel, S. Steinhauer, M. Sowwan, A. Kuronen, K. Nordlund, F. Djurabekova, *ACS Nano* **2016**, 10, 4684.
